# Foreign Summary

**Published:** 1839-12-07

**Authors:** 


### FOREIGN SUMMARY.

*On Hydrated Peroxyde of Iron* (*Ferri Subcarbonas*) *as an Antidote to Arsenic.* By a commission of the Academy of Medicine of Paris.—[In our First, Fourth, and Seventh Volumes, some accounts were published of the facts which seemed to prove the value of peroxyde of iron as an antidote to arsenious acid. The case in the Seventh Volume was one in which a patient that had taken arsenic was rescued from apparently certain death by the administration of this compound by Dr. Deville; and the subject was then deemed of such importance by the Academy of Medicine of Paris, that a commission was appointed, composed of MM. Deville, Sandras, Nonat, and Guibourt, to inquire into the value of the supposed antidote, and the best means of preparing and administering it. The following is an abstract of the therapeutical part of the Report which has been presented.]

Dogs were first destroyed by arsenic, to determine the time in which that poison proves fatal when its action is not interfered with, and to obtain a standard by which the beneficial effects of the antidote might be more nearly estimated. Other dogs were killed by ligature of the œsophagus, to determine the influence which that necessary operation would have in their destruction. Four different oxides of iron were then used with different dogs, to whom arsenic had been given. A moist protoxyde, containing 19 per cent. of anhydrous peroxyde, and a black oxide containing 7 per cent. were entirely inefficacious; the dogs died as soon as they would if no means had been used to save them. The moist hydrated peroxyde, and the common dry hydrated peroxyde of iron (the subcarbonate of iron of former pharmacopœiæ, and ferri-sesqui-oxydum of the present,) both produced more important results. Ten animals poisoned with arsenic were treated with the first. In the first experiments large doses (half a drachm) of arsenic were given, and comparatively small quantities (four, six, or eight ounces) of the magma of peroxyde of iron; yet all the animals lived many hours longer than when the action of the arsenic was left uncontrolled. In the subsequent experiments, twelve grains of arsenic were given, and from eight to sixteen ounces of the antidote; and all the dogs thus treated lived as long as the ligature of the œsophagus would permit them.

With the second compound (the subcarbo nate or sesqui-oxyde of iron,) in three experiments, in which four grains of arsenious acid and three ounces of the antidote were given, the animals, like those last mentioned, lived to the full period which is possible after tying the œsophagus. The arsenite of peroxyde of iron (the salt which was supposed to be found in the stomach in the preceding cases) was next given to dogs, and evidently acted as a virulent poison. But the apparent anomaly of these cases was proved, by some further experiments, to result from the arsenite of iron, thus administered, being decomposed by the hydrochloric and lactic acids of the gastric secretion, and some uncombined arsenious acid being set free; and this showed that, to counteract the effects of the arsenic, it would be necessary to administer the peroxyde of iron in considerable excess, so that every portion of arsenious acid, whether originally existing in the stomach or set free by the decomposition of arsenite of iron, might be completely neutralized.

The results of all these experiments on animals were completely confirmed by the chemical examinations by which M. Guibourt determined the composition and the mutual actions of the substances employed. Thus the first two oxydes that were employed were of no avail against the effects of the arsenic; and from M. Guibourt's chemical researches it was evident that they did not render arsenious acid insoluble. In the physiological experiments the moist hydrated peroxyde of iron neutralized the arsenious acid in the stomach, imperfectly, indeed, when there was a proportionally large quantity of acid, but completely when the proportion of arsenic was small and that of the peroxyde considerable; and in the chemical experiments it appeared that ten parts of the dry peroxyde were necessary to neutralize one of arsenious acid. In the physiological experiments, again, the dry peroxyde completely neutralized the arsenious acid; and in the chemical the same peroxyde reduced to traces scarcely perceptible the precipitate of arsenious acid that could be obtained by Marsh's apparatus.

It is evident, then, (the Report continues,) that in the circumstances which have been determined, dogs have taken arsenic sufficient to destroy them in a few hours, and that yet they have lived as long as if only the œsophagus had been tied. The poison, therefore, has had no effect;—the peroxyde of iron, which we have employed, is, consequently, a real and efficacious antidote to arsenious acid.

Now, it is demonstrated that we have *disempoisoned* dogs that had taken arsenic; we have determined what doses of hydrated peroxyde of iron are necessary to neutralize fatal quantities of arsenious acid; we have employed, with the greatest success, not only the moist peroxyde of iron (always an inconvenient compound,) but also the dry hydrated peroxyde, so common in the shops under the name of subcarbonate of iron, and so easy to administer in all imaginable doses, and so little injurious that we can see no necessity to limit the employment that may be made of it in these cases of poisoning. The following, then, is the mode of treatment which we should advise to be employed in these cases.

The first duty of the physician is to remove from the stomach the greatest possible quantity of the poison, by vomiting. For this purpose watery drinks are improper; but while waiting for the peroxyde of iron, the doctor should tickle the uvula, and'administer oil, which will not dissolve the arsenious acid ; but above all, as soon as it can be procured, the patient should be gorged with warm water, charged with some ounces of the peroxyde. The water will cause vomiting, and the peroxyde of iron, which is suspended in it, will neutralize the particles of arsenious acid that are dissolved. This means fulfils at once the two chief indications in every case of poisoning. The antidote must be given as soon as possible, and in such a quantity and for such a time that there can be no reason to suppose a single atom of arsenious acid remains in the stomach. Four ounces of the dry hydrated peroxyde of iron (the subcarbonate of iron of the shops, the ferri-sesquioxydum of the London Pharmacopœia,) should be suspended in twenty-four ounces of water, and a good glass of the mixture should be taken every ten minutes. After four ounces are consumed, fresh doses of the same compound should be administered in the same way, and the patient should not be considered out of danger till he has taken at least half an ounce of the peroxyde for each grain of arsenious acid supposed to have remained in the stomach. If, afterwards, symptoms of inflammation should manifest themselves, prompt recourse must be had to antiphlogistics, and other appropriate means.

In the short discussion which followed the reading of the Report, M. Devergie said he thought the conclusion too explicit ; that the large quantities of the peroxyde which it was necessary to administer, even when but a small quantity of arsenic had been taken, would always be an obstacle to its use, and that it would be unwise to let the presumed antidote take the place of any of the other means usually resorted to, especially continued vomiting. At the conclusion of the discussion it was agreed to employ the words " anti-dote," instead of "real and efficacious antidote ;" and the Report was then unanimously ordered to be printed.—*Brit. and For. Med. Rev.*, *from Revue Médicale.*

---

*New sign of Death by Hanging.* By ALPH. DEVERGIE.—(*Annales d'Hygiene Publique*, January 1839.)—M. Devergie proposes two additional tests, which he thinks will be of much service to the medical jurist in cases where there exists any dubiety as to whether a body found hanged has been so during life or after death. He has found in all cases of death by hanging "congestion of blood in the organs by which the generative act is accomplished," and "the existence of spermatic animalculæ in the urethral canal." The first of these has been frequently remarked before ; but the last merits notice. He has been able to detect, with the aid of the microscope, spermatic animalculæ both in the semen which has been ejected upon the linen of the deceased, and also in the portions of that fluid still remaining within the canal of the urethra. He has even detected these animalculæ in seminal stains which had been on linen for no less a period than six months ; but in this last case many of the animalculæ were found to have lost their tails, in consequence of the manipulations preparatory to their being subjected to the powers of the microscope.

He suggests the examination of spermatic stains by the microscope as a test of far greater value than any which chemistry is able to furnish. He advises that the fluid be pressed out from the uretha in preference to laying that canal open, as in this way the fallacy which might arise from the intermixture of any portions of blood is avoided. The fluid expressed is placed between two plates of glass and subjected to the microscope, when the spermatic animalculæ, if present, may at once be detected.—*Edinburgh Medical and Surgical Journal, Oct.* 1839.

---

*Muscular Coats of the Excretory Ducts.*—G. H. Myers has published an examination of the muscular layer of the excretory ducts of glands. He used for this purpose a galvanic column of 50 pairs of plates. The gall bladder contracted between the wires to a fourth of its previous diameter ; the ureters also evidently contracted, and the vasa deferentia and vesiculæ seminales, both with galvanic and mechanical stimuli.

In the gall bladder of the sheep and the ureters of the horse there are different layers of reddish-yellow fibres, which in microscopic characters agree exactly with the organic muscular fibres. Their course appears more evidently after they have been boiled. After 24 hours' boiling some gelatine is obtained, probably from the cellular tissue and the remains of the mucous membrane.

The ureters consist of three muscular layers—an external and an internal, of longitudinal fibres, and a layer between them of circular fibres ; the external layer arises from the urinary bladder with strong fasciculi, which become finer towards the kidneys. In the middle circular layer, the fibres lie closer together, and have a resemblance to the middle arterial coat, though they are not of an elastic nature. The fasciculi of the inner layer are more connected, and project somewhat internally.

The gall-bladder receives two fasciculi of long intestinal fibres from the intestine, which pass into its outer layer; below these there is a second series of stronger circular fibres which again covers an obliquely placed layer. Lastly, there is the undermost layer, which consists of longitudinal fibres. It is the same in the shark ; two muscular fasciculi, ascending from the intestine to the ductus choledochus, divide on the gall-bladder, and part ascend even up the hepatic duct. —*Lond. Med. Gaz. from Müller's Archiv. Jahr.*

